# *Cystobasidium alpinum* sp. nov. and *Rhodosporidiobolus oreadorum* sp. nov. from European Cold Environments and Arctic Region

**DOI:** 10.3390/life8020009

**Published:** 2018-05-05

**Authors:** Benedetta Turchetti, Laura Selbmann, Nina Gunde-Cimerman, Pietro Buzzini, José Paulo Sampaio, Polona Zalar

**Affiliations:** 1 Department of Agricultural, Food and Environmental Sciences & Industrial Yeasts Collection DBVPG, University of Perugia, 06121 Perugia, Italy; pietro.buzzini@unipg.it; 2 Department of Ecological and Biological Sciences, University of Tuscia, 01100 Viterbo, Italy; selbmann@unitus.it; 3 Italian National Antarctic Museum, Section of Mycology, 16128 Genoa, Italy; 4 Department of Biology, Biotechnical Faculty, University of Ljubljana, Večna pot 111, SI-1000 Ljubljana, Slovenia; nina.gunde-cimerman@bf.uni-lj.si (N.G.-C.); polona.zalar@bf.uni-lj.si (P.Z.); 5 UCIBIO-REQUIMTE, Departamento de Ciências da Vida, Faculdade de Ciências e Tecnologia, Universidade Nova de Lisboa, 2829-516 Caparica, Portugal; jss@fct.unl.pt

**Keywords:** *Cystobasidium alpinum* sp. nov., *Rhodosporidiobolus oreadorum* sp. nov., psychrophilic yeasts, Arctic, Alps, cold-adapted biodiversity

## Abstract

Over 80% of the Earth’s environments are permanently or periodically exposed to temperatures below 5 °C. Cold habitats harbour a wide diversity of psychrophilic and psychrotolerant yeasts. During ecological studies of yeast communities carried out in cold ecosystem in the Italian Alps, Svalbard (Norway, Arctic region), and Portugal, 23 yeast strains that could not be assigned to any known fungal taxa were isolated. In particular, two of them were first identified as *Rhodotorula* sp., showing the highest degree of D1/D2 sequence identity with *Cystobasidum laryngis* accounted to only 97% with the type strain (*C. laryngis* CBS 2221). The other 21 strains, exhibiting identical D1/D2 sequences, had low identity (97%) with *Rhodosporidiobolus lusitaniae* and *Rhodosporidiobolus colostri*. Similarly, ITS sequences of the type strains of the most closely related species (93–94%). In a 2-genes multilocus D1/D2 and ITS ML phylogenetic tree, the studied strains pooled in two well separated and supported groups. In order to classify the new 23 isolates based on phylogenetic evidences, we propose the description of two novel species *Cystobasidium alpinum* sp. nov. and *Rhodosporidiobolus oreadorum* sp. nov.

## 1. Introduction

The cryosphere covers about one-fifth of the surface of the Earth, with substantial seasonal variations and a long-term trend of losses in its area and volume due to climate warming [1]. Currently, the terrestrial cryosphere includes nearly 200,000 glaciers and two ice sheets [2]. Glacier ice, due to subfreezing temperatures, low water activity (aw), low nutrients availability, and high hydrostatic pressure and oxidative stress, represents one of the harshest environments of the cryosphere for living organisms. Despite these inhospitable conditions, glacial habitats harbour a wide diversity of adapted microorganisms [3]. In spite of the concerted efforts over the last 10 years, there are still major gaps in our knowledge about the fungal biodiversity inhabiting the worldwide cold regions; as a consequence, a detailed knowledge of the microbial biodiversity and its functions (hydrolysis of organic macromolecules, ecological role in nutrient cyclers as in situ organic matter decomposers) is fundamental to understand these important ecosystems, whose existence is endangered due to the phenomenon of global warming.

During ecological studies of yeast communities carried out in cold ecosystem in Italy [4,5], Portugal [6], and the Arctic area [7,8,9,10], 23 yeast strains belonging to two new species in the Pucciniomycotina were isolated and characterized. In particular, two of them (DBVPG 10041 and CCFEE 6272) were first identified as *Rhodotorula* sp. showing the highest degree of D1/D2 sequence identity with *Cystobasidum laryngis* (formerly *Rhodotorula laryngis*). The other 21 strains showed three to seven nucleotide differences when compared with the type strains of the most closely related species: *Rhodosporidiobolus lusitaniae* (formerly *Rhodosporidium lusitaniae*) and *Rhodosporidiobolus colostri* (formerly *Rhodotorula colostri*). 

The subphylum Pucciniomycotina (Phylum Basidiomycota, Kingdom Fungi) includes eight classes of unicellular or dimorphic fungi. Twenty-eight genera of yeast have been described since 2015 [11,12,13]. Since some of them were polyphyletic (*Bensingtonia*, *Rhodosporidium*, *Rhodotorula*, *Sporidiobolus*, and *Sporobolomyces*) and molecular phylogeny based on ribosomal DNA sequence analyses was not concordant with their phenotypic characterizations, Wang et al. [14,15] proposed the reorganization of yeasts and related filamentous fungi within Pucciniomycotina, using multigene (seven genes) sequence analyses.

In particular, the genus *Rhodotorula*—previously strongly polyphyletic and distributed along the classes Microbotryomycetes, Cystobasidiomycetes, and Exobasidiomycetes—was reduced to the clade of *Rhodotorula glutinis*. Since the phylogenetic placement of *Rhodotorula svalbardensis* remained uncertain and a robust molecular analysis was not available, the species was kept as *R. svalbardensis* pro. tem. (pro tempore, temporary taxonomic placement). All the remaining *Rhodotorula* species were amended and included in other known or novel genera [15]. 

The genus *Cystobasidium* was emended by Yurkov et al. [16] in order to accommodate a group of closely related asexual species, some of them previously classified in *Rhodotorula*, along the type species, the sexual mycoparasite *Cystobasidium fimetarium*. Species of this genus are often pink pigmented, yeast cells are generally ovoidal to elongate, and the budding is polar. The sexual state of *C. fimetarium* involves the formation of transversely septate basidia, is not observable in culture, and develops only in natural conditions and in the presence of the appropriate fungal host. 

*Rhodosporidiobolus* was at that time described and included nine asexual species formerly classified in *Rhodotorula* and *Sporobolomyces*, and 16 (nine and seven, respectively) sexual stages, previously classified in *Rhodosporidium* and *Sporidiobolus*. Species of this genus can show sexual reproduction characterized by the formation of mycelium with clamp connections and teliospores. Upon germination, teliospores form transversely septate basidia. Colonies are generally pink to red and butyrous. 

Based on phylogenetic evidences gained from the construction of a ML multilocus tree, the strains here studied were accommodated in the two novel species, *Cystobasidium alpinum* and *Rhodosporidiobolus oreadorum*, that are herewith described. 

## 2. Material and Methods 

### 2.1. Sampling and Isolation

The 23 strains considered were isolated during four different sampling campaigns. Strain DBVPG 10041 was isolated from supraglacial sediment of Miage glacier, Mont Blanc, Italian Alps, Italy. Sampling and isolation was described by Turchetti et al. [4]. Strain CCFEE 6272 was isolated from rock sampled in Pizzo Dosdè, Italian Alps, Italy, at 3280 m asl. Sampling and isolation were carried out as described in Selbmann et al. [5].

All EXF (19) strains were isolated from subglacial ice sampled in Norway, Spitsbergen archipelago, Svalbard, Kongsfjorden, Kongsvegen glacier in July 2008. Samples of glacial ice were collected in duplicates at three separate locations: N 78° 53, 321/E 12° 21, 699 (samples No. 20 and 21), N 78° 53, 973/E 12° 22, 093 (samples No.23 and 24), and N 78° 57, 809/E 12° 26, 947 (samples No. 26 and 27). Ice samples were transported to the laboratory of Natural Environment Research Council Arctic research base, where they were melted aseptically at room temperature, and processed as described by Gunde-Cimerman et al. [7], on the following culture media: synthetic nutrient agar (SNA), malt extract agar (MEA), MEA supplemented with 5% NaCl (MEA5%), malt yeast extract with 20% glucose (MY20%); all culture media were supplemented with 50 mg/L of chloramphenicol. Incubation temperatures were 5 °C and room temperature. 

Strains ZP 292 and ZP 295 were isolated form soil covered by snow in the mountain of Serra da Estrela in Portugal [6].

Strains DBVPG 10041 and CCFEE 6272 are preserved in the Industrial Yeasts Collection DBVPG of the University of Perugia, Italy (http://www.dbvpg.unipg.it), and in the Culture Collection of Fungi from Extreme Environments (Mycological Section of the Italian Antarctic Museum, MNA), University of Tuscia, Viterbo, Italy, respectively, as lyophilized (4 °C) and long-term cryopreserved (−80 °C) cultures. Strains with EXF acronym are preserved at −80 °C in the Culture Collection of Extremophilic Fungi Ex (http://www.ex-genebank.com/index.php/en/fungi-2), at the University of Ljubljana, Slovenia. Strains PYCC 8103 and PYCC 8104 are conserved at the Portuguese Yeast Culture Collection (PYCC), Universidade Nova de Lisboa, Portugal.

### 2.2. Physiology

Physiological and biochemical tests were performed according to the protocols described by Kurtzman et al. [17] using the following media: Malt Extract broth (ME, Oxoid), Malt Extract Agar (MEA, Oxoid), Potato Dextrose Agar (PDA, Oxoid), and Corn Meal Agar (CMA, Difco). Before performing the tests the optimum temperatures (defined as the temperature at which the maximum growth was detected) of both species were determined using MEA solid medium [17], inoculating spots of 5 μL of decimal dilutions of each strains. The Petri dishes were incubated at different temperatures (10 °C, 12 °C, 15 °C, 18 °C, 20 °C, 25 °C). The test was performed in triplicate. The temperature at which the most abundant growth at the maximum dilution was observed, was defined as the optimum temperature. Physiological and biochemical tests were done in duplicate at 15 °C for both *C. alpinum* and *R. oreadorum*. Results were recorded after 2 and 4 weeks of incubation. Results obtained from duplicate experiments were concordant. 

### 2.3. Morphology

For microscopy, cultures were grown on PDA agar at room temperature and studied with a Nikon Microphot-FX microscope and Olympus BX51 microscope, the latter equipped with differential interference contrast optics. Images of colony morphology were taken using a stereo-microscope equipped a Nikon zoom digital camera, COOLPIX950. For determination of sexual compatibility, pairs of 7-day-old cultures were crossed on corn meal agar, incubated at 16–18 °C for one week and examined for production of mycelium and teliospores after 1–6 weeks. To induce teliospore germination, 6-week-old agar blocks containing them were soaked in demineralized water for 8 weeks at 4 °C. Then the teliospores were transferred to 2% water agar at 20–22 °C.

### 2.4. Molecular Analysis and Phylogeny

The D1/D2 domains of the large subunit (28S) ribosomal gene region and the Internal Transcribed Spacer region (ITS1 and 2) including the 5.8S rRNA gene were amplified using standard primers (NL1 and RLR3R for LSU; ITS1 and ITS2 for ITS regions) [18]. Amplifications were performed using the T personal Combi Thermal Cycler (Biometras Gmbh, Goettingen, Germany), applying amplification protocols reported by Selbmann et al. [5]. Sequences were obtained with an ABI PRISM 3730XL Capillary Sequencer using the standard protocol recommended by the manufacturer. ITS and D1/D2 sequences were blasted in the NCBI database. The most similar sequences were exported, alignments were generated using MAFFT version 6 [19] and improved manually in MEGA6 [20]. ITS and D1/D2 alignments were concatenated using FABOX (http://users-irc.au.dk/biopv/php/fabox/alignment_joiner.php, [21]). Combined sequences of two-locus datasets alignments were exported and the best-fit substitution model was determined using MODELTEST MrAIC 1.4.3 [22] as implemented in PHYML [23].

Phylogenetic ML trees reconstruction was performed selecting the best substitution model gained with MODELTEST MrAIC 1.4.3 in MEGA6. The robustness of the phylogenetic inference was estimated using the bootstrap method [24] with 1000 pseudoreplicates generated and analyzed with TREEFINDER.

## 3. Results 

The complete list of the strains, their isolation sources, and their sequences accession numbers are shown in Table 1. Phylogeny was based on the two-gene dataset that included 42 strains which sequences could be confidently aligned with the ones of studied strains; the dataset was based on 1348 positions (ITS: 1–707; D1/D2: 708–1, 348) including gaps.

**Figure 1:**
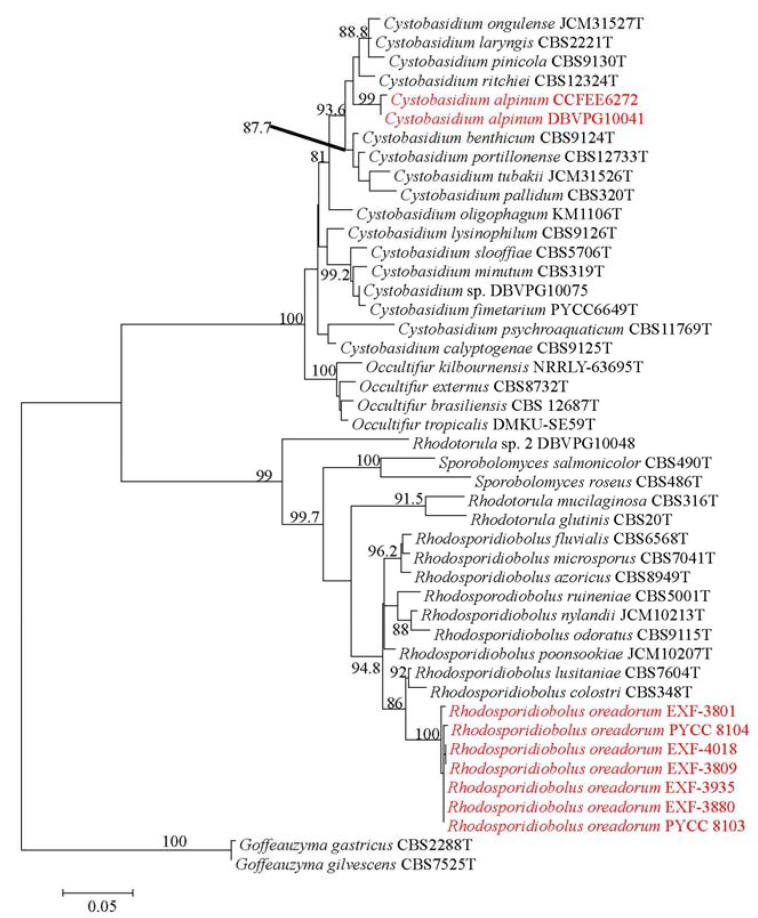
ML 2-genes multilocus phylogeny (ITS and D1/D2) showing the phylogenetic placement of the new taxa described. The tree, based on 42 strains and 1348 nucleotide positions, has been generated using a GTR+IG(4) model calculated using ML in the software MrAIC. Bootstrap values above 80%, calculated from 1000 resampled data sets, are shown.

The resulting multilocus phylogenetic tree, generated using a GTR+G(4) model selected using the Akaike’s information criterion with a Maximum-likelihood approach, is shown in Figure 1. The backbone of the tree and branches of genera are well resolved and supported. The tree topology is concordant with the most recent phylogenetic studies on yeasts in Pucciniomycotina [14] and on the genus *Cystobasidium* in particular [25].

The two strains DBVPG 10041 and CCFEE 6272 pooled in a separated and well supported branch (bootstrap 99%) in the genus *Cystobasidium*, confirming the high divergence observed with BlastN analysis. The D1/D2 sequences were, in fact, only 97% similar to sequences available in the public domain and the closest species for strain DBVPG 10041 was *C. laryngis*, 16 bp different. Surprisingly, a lower divergence with known species was found when the target ITS (1 and 2) sequence was blasted in the public domain, despite this gene being, as barcode for fungi and hence for the genus *Cystobasidium* too, extremely variable [16]. In particular, DBVPG 10041 and CCFEE 6272 have the same ITS sequence that differ by 12 nucleotide substitutions from the type strain of *Cystobasidium pinicola* and 16 with *C. laryngis*. Only 10 nucleotide substitutions differentiate DBVPG 10041 and CCFEE 6272 from two sequences deposited in Genbank as *Rhodotorula* sp. BC22 [26] and Antarctic yeast CBS 8923 (unpublished), both isolated from Antarctica and not yet described. 

Based on these considerations and on the phylogenetic position in the multilocus ML tree, these two strains are here described as new species and *Cystobasidium alpinum* is proposed to accomodate the strains DBVPG 10041 and CCFEE 6272.

The main macroscopic characteristic that distinguishes *C. alpinum* from the closest species is the colour of its colonies: cream to brownish-gray while all the species of *C. laryngis* clade are coloured from light pink to salmon, to red (Table 2). Additionally, optimum growth temperature for *C. alpinum* is 15 °C and maximum 25 °C, while all the closest species had optimum temperature from 15–17 °C to 25 °C, all of them duplicated at 25 °C and, in few cases, even at 30–35 °C (Table 2). Other physiological differences are shown in Table 2. 

The *C. laryngis* clade is mainly composed of species which originate in cold environments. In this clade three new Antarctic species were recently described: *Cystobasidium tubakii*, *Cystobasidium ongulense*, and *Cystobasidium portillonense* [25,28]. Additionally, some strains isolated from a glacier in the Alps, first identified as *Rhodotorula* cf. *laryngis*, were later identified as *Cystobasidium ongulense* [4]. Moreover strains of *C. laryngis*, *C. pinicola*, and *Cystobasidium benticum* were also all isolated from cold habitats: Antarctica, Greenland, cold lake in Argentina, and deep sea. All listed species, including *C. alpinum*, share psychrophilic or psychrotolerant aptitude, showing optimum temperature between 15 °C and 20 °C. The most defining characteristics of *C. laryngis* clade are therefore their origin in cold environment and adaptations to the habitat temperatures. 

The remaining strains listed in Table 1 share similar D1/D2 and ITS sequences. PYCC 8104 had one mismatch in D1/D2 sequences when compared to the other strains. Nucleotide differences in ITS sequences ranged from one to three. The D1/D2 sequence of the type strain EXF-3880 showed three nucleotide substitutions in comparison to the closest species *R. lusitaniae*, and eight substitutions in comparison to *R. colostri*; a larger divergence with 27 and 28 mismatches was found in ITS sequences. The ML multilocus phylogeny placed all these strains in a well-supported (100%) and separate branch, with *R. lusitaniae* and *R. colostri* as closest neighbours. Based on the evidences above, we here allocate these strains in a new species and propose the name *Rhodosporidiobolus oreadorum R. oreadorum* isolates differed from the closest species also in their abilities to assimilate cellobiose and nitrite, in the production of starch like compounds and in the formation of capsule (Table 3). Additionally, after crossing sexually compatible strains of *R. oreadorum*, true hyphae with complete clamp connections were observed together with spherical teliospores formed terminally and intercalarly in the mycelium. The sexual structures of *R. oreadorum* show some differences when compared with those of *R. lusitaniae*, its closest sexual relative. In the mycelium of *R. lusitaniae*, clamp connections are not formed, whereas those structures are formed by *R. oreadorum*. Interestingly, *R. lusitaniae* is homothallic while *R. oreadorum* is heterothallic. Another distinctive feature is the presence of an elongated stalk connecting the basidium to the teliospore in *R. lusitaniae,* whereas such stalk is absent in *R. oreadorum* [29]. Maximum growth temperature for *R. oreadorum* was 30 °C, characterized by weak growth, while all the other species displayed richer growth at the same or higher maximum temperatures (Table 3). Other physiological differences are shown in Table 3. All the Arctic strains of *R. oreadorum* were isolated exclusively from subglacial ice, and not from adjacent samples like ice at the margin of the glacier, stream water on the glacier, glacial melt water, or adjacent sea water. The two strains isolated in Portugal were found in soil under snow in a mountainous region.

## 4. Discussion

It is commonly hypothesized that microbial cells that originate from geographically close and distant places are deposited with snow and gradually become embedded in the ice layers of glaciers or surface of soil covered with snow [4]. After this, the cells concentrate in the veins of liquid water between the forming ice crystals, where the micro-conditions can support the survival of the best adapted species despite the hostile surrounding environment [32].

Microorganisms that grow in such habitats are commonly referred to as psychrophiles, if they are unable to grow at temperature >20 °C, or as psychrotolerant, if they tolerate low temperatures, but have a growth temperature optimum above 15 °C [33].

In recent years, a few studies [8,34] discovered the presence of fungi, including yeasts, beneath and in meltwater draining off from polythermal glaciers, where ice melting occurs due to changes in pressure. Since global warming is predicted to be most pronounced at high latitudes, Arctic and Alpine glaciers are and will be habitats intensively threatened by climate change. 

All the strains of the present study were isolated from cold environments: Svalbard, Italian Alps and soil under snow layer in Portuguese mountains (Table 1). Physiology denounces a cold-adaptation of the strains examined; in fact, they showed a psychrotolerant aptitude, having an optimum growth temperature at 15–18 °C, and weak or no growth at 25 or 30 °C (Table 2 and Table 3). In the past, cryospheric environments were considered abiotic; however, they are now recognised as ecosystems containing an enormous reservoir of microbial diversity, as exemplified by the description of the two new species. Their recognition will improve our understanding of the dynamics of the extremely cold environments and also provide additional crucial information for predicting the consequences of the massive changes of the Arctic glacial ecosystems caused by global climate changes. Additionally, the global warming will change the climatic condition of cold ecosystems and will determine the melting of glaciers. These habitats are now considered in danger of disappearing [35] as well as the psychrophilic microbial biodiversity associate to them. In this context, the isolation, description, and ex situ conservation of psychrophilic microorganisms can be considered a way for avoiding their possible extinction.

### 4.1. Description of the Species

#### 4.1.1. Description of *Cystobasidium alpinum* B. Turchetti, L. Selbmann, S. Onofri & P. Buzzini sp. nov.

*Cystobasidium alpinum* (al.pi.’num N.L. neut. adj., ‘alpinum’ referring to Alps, the mountain complex from which the strains were isolated). 

**Figure 2:**
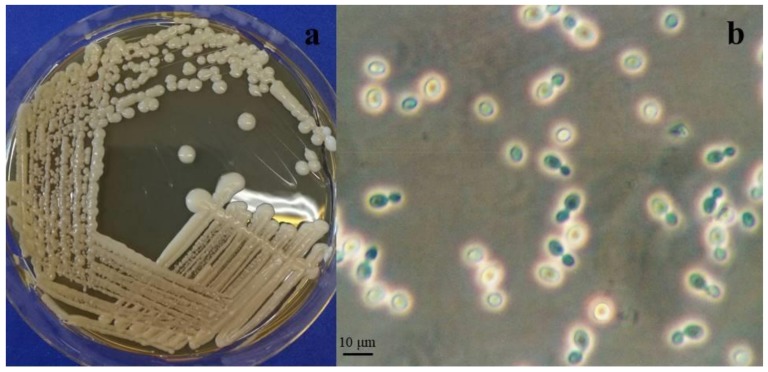
*Cystobasidium alpinum* (**a**) Colonies of DBVPG 10041^T^ on MEA after 2 weeks incubation at 15 °C. (**b**) Polar budding cells of DBVPG 10041^T^ on MEA after 1 week incubation at 15 °C.

Novel yeast species belonging to phylum Basidiomycota, subphylum Pucciniomycotina, class Cystobasidiomycetes, order Cystobasidiales, family Cystobasidiaceae. Telomorph: not observed. MycoBank: MB#824354 (Figure 2).

After 14 days on malt extract agar (MEA), corn meal agar (CMA), and potato dextrose agar (PDA) at 15 °C, the colonies were cream to brownish-gray, smooth and glistening, with entire margin, flat profile, and viscous texture. On YPDA (yeast extract 1%, peptone 1%, dextrose 2%, agar 2%) the colonies were bigger and dull and showed butyrous texture and raised profile. The cells were ovoidal, 2–5 µm long, and 1–2 µm wide, and the budding was polar. In none of the strains, pseudohyphae were observed. Sediment was produced when the strains grew in Malt extract broth (ME) and YPD at 15 °C, after 7 days. After 14 days, a light superficial ring was also present. No positive mating reactions were observed among the two strains using different media.

Glucose was not fermented. Glucose, d-xilose, l-arabinose, a,a trehalose, cellobiose, salicine, arbutin, glycerol, d-mannitol, glucono d-lactone, d-gluconate, succinate, ethanol, *N*-acetyl d-glucosamine, and ethyl acetate were assimilated by both strains. d-galactose, d-arabinose, methyl α-glucoside, melibiose, lactose, raffinose, melezitose, mesoerythritol, ribitol, xylitol, d-glucitol, and l-lactate were weakly assimilated, while no growth was observed on l-sorbose, d-ribose, l-rhamnose, maltose, galacticol, myo-inositol, d-glucuronate, citrate, methanol, and hexadecane. Sucrose and l-malic acid gave different results in the two tested strains, and thus can be considered variably assimilated. Assimilation of nitrogen compounds was positive for l-lysine and ethylamine (HCl), weak for cadaverine and nitrite, and negative for nitrate.

Growth at 10 °C, 15 °C, 20 °C was good, while it was delayed at 4 °C and very weak at 25 °C; no growth was shown at 30 °C. Optimum growth temperature is 15 °C.

Growth YE agar with 50% glucose was absent while was weak and delay on YNB broth with 10% NaCl +5% glucose. Starch-like compounds were not produced. In 0.01% cycloheximide, growth was weak and absent at 0.1% concentration. Urease activity was absent, while Diazonium Blue B reaction was positive. 

The type strain of *Cystobasidium alpinum* sp. nov. was isolated from supraglacial sediments of Miage glacier, Mont Blanc, Italian Alps, Italy (45°84′70” N; 68°52′00” E), and has been deposited in the Industrial Yeasts Collection DBVPG, Department of Agricultural, Food and Environmental Sciences, University of Perugia, Italy, under the codes DBVPG 10041T, and in CBS KNAW Collection as CBS 14809T.

Gene sequence accession numbers of the type strain: D1/D2 LSU rRNA = KC433879, ITS = KC455920. Two strains were reported in the present paper all isolated from cold habitats located in Alps: Mont Blanc and Pizzo Dosdè (Table 1).

#### 4.1.2. Description of *Rhodosporidiobolus oreadorum* P. Zalar, J.P. Sampaio & N. Gunde-Cimerman sp. nov.

*Rhodosporidiobolus oreadorum* (o.re.a.’dum N.L. gen. plu. neut. n., oreadorum, referring to mountain nymphs). 

**Figure 3:**
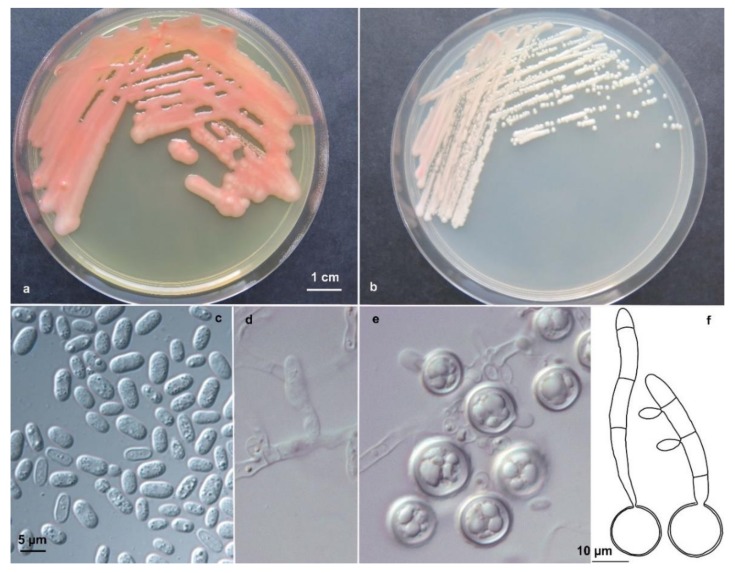
*Rhodosporidium oreadorum* Colonies of EXF-3880^T^ on PDA (**a**) and MEA (**b**) after 1 week of incubation at 20 °C. Polar budding (**c**) of EXF-3880^T^ on MEA after 1 week of incubation at 20 °C. After mating compatible strains on SG agar: mycelium with clamp connections (**d**), teliospores (**e**), germinated teliospores with transversely septate basidia and basidiospores (**f**). Scale bar indicated on figure (**c**) is also valid for figures (**d**) and (**e**).

Novel yeast species is belonging to the phylum Basidiomycota, subphylum Pucciniomycotina, class Microbotryomycetes, order Sporidiobolales, family Sporidiobolaceae. Telomorph: production of basidia from germinated teliospores. MycoBank: MB#824286 (Figure 3).

After 7 days on malt extract/yeast extract agar at 18 °C, the colonies are reddish, smooth and shiny, with an entire margin. On PDA, the red colony color was more intense. The cells are ovoidal to ellipsoidal, 3–9.5 µm long, in average 5.8 µm (SD 1.3) and 2.5–5 µm wide, in average 3.3 µm (SD 0.7), with a capsule, and they reproduce by bilateral budding. In Dalmau plates after 2 weeks on cornmeal agar, pseudohyphae and true hyphae are not formed in single strains. Heterothalic, after crossing sexually compatible strains on CMA or SG agar true hyphae, 1 µm in diameter, with complete clamp connections and terminal as well as intercalary teliospores, 14–18 µm in diameter, developed after one week. Teliospores germinated after a resting stage of 6 weeks at 4 °C and formed transversely septate basidia with four compartments. Basidiospores were ovoidal and measured 5–6 × 2–3 µm. The germination of teliospores was observed in a cross involving strains from Portugal.

Glucose was not fermented. Glucose, sucrose, d-galactose, d-ribose (weak), d-xilose, l-arabinose, trehalose, raffinose (weak), fructose, ribitol, glycerol (slow), d-mannitol, succinate, citrate, ethanol, and sorbitol were assimilated. No growth occured on d-glucosamine, l-rhamnose, maltose, cellobiose, melibiose, lactose, melezitose, d-glucuronate, myo-inositol, soluble starch, l-rhamnose, methanol, lactate, and hexadecane. Assimilation of nitrogen compounds was positive for nitrate, no growth was observed on nitrite and l-lysine.

Growth in vitamin-free medium was positive. Growth at 5–25 °C was good, weaker on 30 °C; no growth at 37 °C. Growth on YM agar with 5% NaCl, no growth with 10% NaCl. Starch-like compounds were produced. In 100 μg mL^−1^ cycloheximide, growth was absent. Urease activity is positive. Diazonium Blue B reaction is positive.

The type strain of *Rhodosporidium oreadorum* sp. nov. was isolated from subglacial ice in Kongsvegen glacier (N 78° 53, 973; E 12° 22, 093) in Kongsfjorden, Svalbard, Norway and has been deposited in the Culture Collection of Extremophilic Fungi (Ex), Department of Biology, Biotechnical Faculty, University of Ljubljana, Slovenia under the accession number EXF-3880T. The isotype was submitted to CBS culture collection as CBS 14900^IT^, and to the Portuguese Yeast Culture Collection (PYCC), Universidade Nova de Lisboa, Portugal, as PYCC 6772. Gene sequence accession numbers of the type strain: D1/D2 28S rRNA = MF189940, ITS = MF189945.

Complementary mating types: strains EXF-3820 (PYCC 6771), EXF-3933 (PYCC 6773), EXF-3935, EXF-3969, and PYCC 8103 belong to mating type A1, while EXF-3880, EXF-3881, EXF-3936, EXF-3970, EXF-3986, EXF-4018 (PYCC 6774), EXF-4228, EXF-4230, EXF-4235, and PYCC 8104 belong to mating type A2.

Additional strains reported in the present paper, were isolated all from subglacial ice in Svalbard, Kongsfjorden: EXF-3809 (28S rRNA = MF189941; ITS = MF189946), EXF-3801 (28S rRNA = MF189942; ITS = MF189947), EXF-3935 (28S rRNA = MF189943; ITS = MF189948), and EXF-4018 (28S rRNA = MF189944; ITS = MF189949.

Strains PYCC 8103 (ZP 292) and PYCC 8104 (ZP 295) were isolated from soil under snow, in Serra da Estrela mountain, Portugal (PYCC 8103, 28S rRNA = JN246537, ITS = JN246560; PYCC 8104, 28S rRNA = JN246538, ITS = JN246561).

## 5. GeneBank Numbers of the Type Strains

*Cystobasidium alpinum*: DBVPG 10041T = CBS 14809T (D1/D2 28S rRNA = KC433879, ITS = KC455920); *Rhodosporidiobolus oreadorum*: EXF-3880T = CBS 14900IT (D1/D2 28S rRNA = MF189940, ITS = MF189945).

## Figures and Tables

**Table 1:** List of studied strains, their origins, and GenBank accession numbers.

Species	Strain Numbers		Isolation Source	Sample	GenBank Accession Numbers	
					D1/D2 28S rDNA	ITS rDNA
*Cystobasidium alpinum*	DBVPG 10041^T^	CBS 14809^T^	Miage glacier, Mont Blanc, Italian Alps, Italy (45°84′70” N; 68°52′00” E)	supraglacial sediments	KC433879	KC455920
	CCFEE 6272		P.zzo Dosdè, Cima di Piazzi group, Italian Alps, Italy	rock	KY626311	KY626312
*Rhodosporidiobolus oreadorum*	PYCC 8103 (ZP 292)		Serra da Estrela, Portugal	soil under snow	JN246537	JN246560
	PYCC 8104 (ZP 295)		Serra da Estrela, Portugal	soil under snow	JN246538	JN246561
	EXF-3880^T^	CBS 14900^IT^, PYCC 6772^IT^	Kongsvegen glacier (N 78° 53,973; E 12° 22,093), Svalbard, Norway	subglacial ice	MF189940	MF189945
	EXF-3661		(N 78° 53, 973; E 12° 22, 093)	subglacial ice with sediment	-	-
	EXF-3809		N 78° 53, 973; E 12° 22, 093	subglacial ice	MF189941	MF189946
	EXF-3820	PYCC 6771	(N 78° 53, 973; E 12° 22, 093),	subglacial ice with sediment	-	-
	EXF-3881		(N 78° 53, 973; E 12° 22, 093)	subglacial ice with sediment	-	-
	EXF-3884		(N 78° 53, 973; E 12° 22, 093)	subglacial ice with sediment	-	-
	EXF-3801		(N 78° 53, 973; E 12° 22, 093)	subglacial ice	MF189942	MF189947
	EXF-3927		(N 78° 053, 321; E 012° 21, 699)	subglacial ice	-	-
	EXF-3933	PYCC 6773	(N 78° 053, 321; E 012° 21, 699)	subglacial ice	-	-
	EXF-3934		(N 78° 053, 321; E 012° 21, 699)	subglacial ice		
	EXF-3935		(N 78° 053, 321; E 012° 21, 699)	subglacial ice	MF189943	MF189948
	EXF-3936		(N 78° 053, 321; E 012° 21, 699)	subglacial ice	-	-
	EXF-3969		(N 78° 57, 809; E 012° 26, 947)	subglacial ice	-	-
	EXF-3970		(N 78° 57, 809; E 012° 26, 947)	subglacial ice	-	-
	EXF-3986		(N 78° 57, 809; E 012° 26, 947)	subglacial ice		
	EXF-4018	PYCC 6774	(N 78° 57, 809; E 012° 26, 947)	subglacial ice	MF189944	MF189949
	EXF-4230		(N 78° 57, 809; E 012° 26, 947)	subglacial ice	-	-
	EXF-4235		(N 78° 57, 809; E 12° 26, 947)	subglacial ice with white vessels	-	-
	EXF-4228		(N 78° 053, 321; E 012° 21, 699)	subglacial ice	-	-

**Table 2:** Physiological characteristics of the species *Cystobasidium alpinum* and closest related species.

Species	Cyst. alpinum ^a^	Cyst. laryngis ^b^	Cyst. tubakii ^c^	Cyst. ongulense ^c^	Cyst. pinicola ^b^	Cyst. ritchiei ^d^	Cyst. benthicum ^b^	Cyst. pallidum ^b^	Cyst. portillonense ^e^
**Optimum temperature [°C]**	**15**	20	15–17	20	20	20–22	20	20	25
**Colonies colour**	**cream to brownish-gray**	light pink	light pinkish	light pinkish	light pink	red	light pink	pale pink	salmon-pink
**Production of hyphae or pseudo hyphae**	**no**	no	no	no	no	no	no	no	no
**Carbon compounds**									
d-galactose	**-**	-/d	w	-	+	-	+	-	w
d-ribose	**-**	-	-	-	w	+	+	+	-
d-arabinose	**v**	+	-	+	+	+	+	-	-
l-rhamnose	**-**	-	-	-	w	-	+	-	-
Sucrose	**v**	+	+	+	-	+	+	-	-
Maltose	**-**	-	-	-	w	-	+	-	-
Methyl a-glucoside	**w**	-	-	-	-	-	+	-	-
Cellobiose	**+**	+	w	-	w	+	w	-	+
Salicin	**+**	+	+	+	w	+	-	-	w
Raffinose	**w**	-	-	-	w	-	+	-	-
Melezitose	**w**	+	+	+	+	+	+	-	+
d-glucitol	**w**	+	-	+	-	+	-	w/s	+
d-mannitol	**+**	+	-	+	+	+	-	+	+
**Nitrogen coumpounds**									
Nitrite	**w**	-	nd	nd	-	-	-	-	-
Ethylamine	**+**	nd	nd	nd	nd	-	nd	nd	-
l-lysine	**+**	nd	nd	nd	nd	-	nd	nd	w
Cadaverine	**w**	nd	nd	nd	nd	-	nd	nd	-
**Other tests**									
Urea test	**-**	nd	nd	nd	nd	nd	nd	nd	+
Cycloheximide 0.01%	**+/d**	w/s	-	-	-	nd	+	-	-
Growth at 25 °C	**w**	+	+	+	+	+	+	+	+
Growth at 30 °C	**-**	v	-	+	+	-	+	+	+
Growth at 35 °C	**nd**	-	nd	nd	-	nd	+	-	-
Growth at 37 °C	**nd**	nd	nd	nd	nd	nd	+	nd	nd

+: growth; -: no growth; d: delayed growth; w: weak growth; s: slow growth; v: variable; nd: not determined. ^a^ Data from the present study; ^b^ Data obtained from Sampaio [27]; ^c^ Data obtained from Tsuji et al. [25]; ^d^ Data obtained from Yurkov et al. [16]; ^e^ Data obtained from Laich et al. [28].

**Table 3:** Physiological characteristics of the species *Rhodosporidiobolus oreadorum* and closest related species.

Species	Rhodo. oreadorum ^a^	Rhodo. colostri ^b^	Rhodo. lusitaniae ^c^	Rhodo. azoricus ^c^	Rhodo. fluvialis ^c^	Rhodo. nylandii ^d^	Rhodo. microspores ^e^	Rhodo. poonsookiae ^d^
**Optimum temperature [°C]**	**18**	20	20	20	20	17	20	17
**Colonies colour**	**reddish**	deep pink	pink	light orange	deep pink,	deep orange to reddish-orange	light pink	reddish orange
**Production of hyphae or pseudo hyphae**	**heterothalic, true hyphae with clamp connections and intercalary or terminal teliospores**	Poorly developed pseudohyphae	self-fertile, true hyphae with clamp connections and intercalary or terminal teliospores	Poorly developed pseudohyphae	and true hyphae bearing teliospores	Pseudohyphae and true hyphae + ballistoconidia	True hyphae with clamp connections + ballistoconidia	Pseudohyphae and true hyphae + ballistoconidia
**Sexual reproduction**	**yes–teliospores and clamp connections**	no	yes–teliospores and clamp connections	yes–teliospores and clamp connections	yes–teliospores and incomplete clamp connections	no	yes–teliospores and clamp connections	no
**Carbon compounds**								
C 2 d-galactose	**+**	s/w	+	+	+	-	+	d
C 5 d-ribose	**w**	-	-	+	+	d/w	+	+
C 6 d-xylose	**+**	v	+	+	+	-	+	+
C 7 l-arabinose	**+**	-	-	+	+	-	+	+
C10 sucrose	**+**	+/s	-	+	+	+	+	-
C11 maltose	**-**	s/w	-	+	+	+	-	-
C14 cellobiose	**-**	w/s	v	+	+	+	+	+
C19 raffinose	**w**	-	-	+	+	+	s	-
C20 melezitose	**-**	v	-	-	+	+	-	-
C40 citrate	**+**	+/s	+	+	w/s	-	+	w
**Nitrogen coumpounds**								
Nitrate	**+**	+	+	+	+	+	+	+
Nitrite	**-**	+	+	+	+	+	+	+
**Other tests**								
Starch like compounds	**+**	-	-	-	-	-	-	-
Vitamin-free	**+**	-	+	-	+	+	+	+
Cycloheximide 0.01%	**-**	-/w	-	+	w/s	nd	+	nd
Growth at 25 °C	**+**	+	+	+	+	+	+	+
Growth at 30 °C	**w**	v	+	+	+	+	+	+
Growth at 35 °C	**nd**	-	-	-	+	+	-	+
Growth at 37 °C	**-**	nd	nd	nd	+	-	nd	-

+: growth; -: no growth; d: delayed growth; w: weak growth; s: slow growth; v: variable; nd: not determined. ^a^ Data from the present study; ^b^ Data obtained from Sampaio [27]; ^c^ Data obtained from Sampaio [29]; ^d^ Data obtained from Hamamoto et al. [30]; ^e^ Data obtained from Sampaio [31].
